# A cross-sectional descriptive study to assess the impact of the “open door” policy on patient satisfaction

**DOI:** 10.1192/j.eurpsy.2023.1927

**Published:** 2023-07-19

**Authors:** M. Campillo, J. Marti, L. Rius, S. Garcia Fernandez, M. Olivero, G. Sanchez Tomico, G. Brusco-Passalaqua, E. Pechuan, T. Vates, R. Sanchez

**Affiliations:** Psychiatry, Parc de Salut Mar, Santa Coloma Gramenet, Spain

## Abstract

**Introduction:**

Since the beginning of the modern psychiatry the acute units have established a “locked door” policy. Some studies show that this condition may increase patient’s discomfort and affect the perception of health quality of care (Boyer L, 2009, Eur Psychiatry Dec;24(8):540-9). Lately, several European countries such as Germany, Switzerland and Spain are starting to implement the “open-door” policy but its impact on patient’s satisfaction is still unknown (Hochstrasser, L, Frontiers in Psychiatry, 9(57). https://doi.org/10.3389/fpsyt.2018.00057) .

**Objectives:**

To help characterize the advantages of the “open-door” policy implemented in an acute inpatient psychiatric unit in order to assess the patient’s view of it.

**Methods:**

This is a descriptive observational study carried out at an inpatient psychiatric unit. Data were collected after the implementation of the open door policy on June 2019, assessing the patient satisfaction of 31 subjects who completed the SATISPSY-22 scale at the time of discharge. Results are described using the average and its standard deviation.

**Results:**

Results show scores in all items above 50 points, being the care team and the quality of care the most valued ones with 82 and 79 points respectively. The overall score is above 65 points (Fig. 1).

**Image:**

**Figure:**
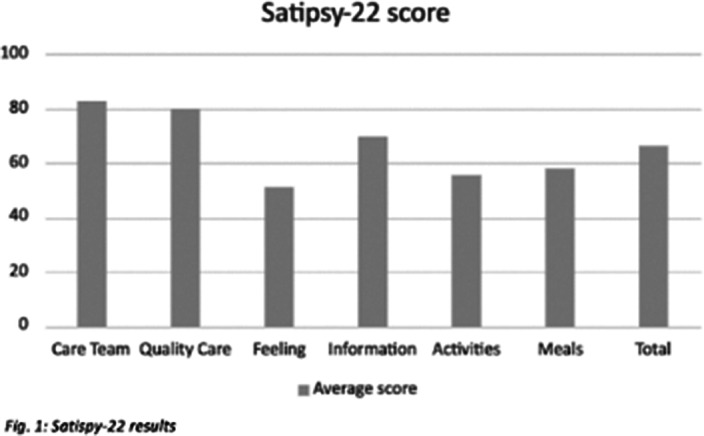

**Conclusions:**

In line with previous studies, our data suggests that the main impact of the “open-door” policy implementation is on patients’ perception of the care, being Quality of care and satisfaction with the Staff the items with highest scores. This could be explained by patients trusting more in the Care team, which would help enhance the therapeutic relationship improving therapeutic adherence, treatment adequacy and the outcome. Nevertheless, the Feeling related to hospitalisation was found to be the item with the lowest score. This could mean strategies should focus on improving patient’s insight regarding their clinical state and their need to be admitted. Our study supports the hypothesis that open-door policy in acute psychiatric units is seen positively by patients and that further research should be carried.

**Disclosure of Interest:**

None Declared

